# Characterization of tea (*Camellia sinensis* L.) flower extract and insights into its antifungal susceptibilities of *Aspergillus flavus*

**DOI:** 10.1186/s12906-023-04122-5

**Published:** 2023-08-14

**Authors:** Fangfang Chen, Yu-Pei Chen, Hongtan Wu, Ya Li, Shudi Zhang, Jincheng Ke, Jeng-Yuan Yao

**Affiliations:** 1 Department of Public Health and Medical Technology, Xiamen Medical College, Xiamen, Fujian 361023 China; 2 Engineering Research Center of Natural Cosmeceuticals College of Fujian Province, Xiamen Medical College, Xiamen, Fujian 361023 China; 3 School of Public Health, Fujian Medical University, Fuzhou, Fujian Province China; 4 Department of Dermatology, The Second Affiliated Hospital of Xiamen Medical College, Xiamen, Fujian 361000 China; 5 Department of Basic Medicine, Xiamen Medical College, Xiamen, Fujian 361023 China

**Keywords:** *Camellia sinensis* L., Tea flower, Antifungal activity, *Aspergillus flavus*, 2-Ketobutyric acid

## Abstract

**Background:**

Tea (*Camellia sinensis* L.) flowers will compete with tea leaves in nutrition and are abandoned as an undesirable by-product. In this study, the biological efficacy of tea flowers was investigated. Further exploration of its antifungal activity was explained.

**Methods:**

Tea flowers harvested from China were characterized in term of component, antioxidant ability, tyrosinase inhibition, and antifungal ability. Chemical compounds of tea flowers were analyzed by LC-MS. Disinfectant compounds were identified in tea flowers, and 2-ketobutyric acid exhibited antifungal activity against *Aspergillus flavus*CCTCC AF 2023038. The antifungal mechanism of 2-ketobutyric acid was further investigated by RNA-seq.

**Results:**

Water-soluble tea flower extracts (TFEs) exhibited free radical scavenging activity against 2,2-diphenyl-1-picrylhydrazyl (DPPH) and 2, 2’-azino-bis(3-ethylbenzothiazoline-6-sulfonic acid)(ABTS) as well as a high ferric-reducing ability. However, no inhibition of tyrosinase activity was observed. In the antifungal test, 6.4 mg/mL TFE reached 71.5% antifungal rate and the electrical conductivity of the culture broth increased with increasing concentration of TFE, implying that it damaged the fungal cell membrane by the TFE. Several disinfectants were identified in TFE by LC-MS, and 2-ketobutyric acid was also confirmed to be capable of fungal inhibition. Propidium iodide (PI) staining indicated that 2-ketobutyric acid caused damage to the cell membrane. RNA-seq analysis revealed that 3,808 differentially expressed genes (DEGs) were found in *A. flavus* CCTCC AF 2023038 treated by 2-ketobutyric acid, and more than 1,000 DEGs involved in the integral and intrinsic component of membrane were affected. Moreover, 2-ketobutyric acid downregulated aflatoxin biosynthesis genes and decreased the aflatoxin production.

**Conclusions:**

Overall, TFE exhibited excellent antioxidant ability and fungal inhibition against *A. flavus* CCTCC AF 2023038 due to its abundant disinfectant compounds. As a recognized food additive, 2-ketobutyric acid is safe to use in the food industry and can be utilized as the basis for the research and development of strong fungicides.

**Supplementary Information:**

The online version contains supplementary material available at 10.1186/s12906-023-04122-5.

## Background

Tea (*Camellia sinensis* L.), a woody plant with a life span of over 100 years, is cultivated in many Asian countries such as China, India, and Japan. Its leaves are globally consumed as a beverage because of its favorable flavor and health benefits for human beings [1]. Much attention has been devoted to the biological activities and beneficial health effects of tea. Studies have demonstrated the anticancer, neuro-protection, cardio-protection, gastro-protection, anti-diabetes, anti-obesity, low-density lipoprotein oxidation reduction, and osteoporosis prevention functions of tea [2]. Tea leaves contain biological components, such as polyphenols, catechins, gallocatechin, epicatechins, gallic acid esters, theaflavins, L-theanine, caffeine, and amino acids, which have been investigated also [2, 3].

Tea flower is a natural and abundant resource with an annual yield of over 4 million tons in China [4]. Tea flower competes with tea leaves in nutrition and is abandoned as an undesirable by-product. However, tea flower can be used as traditional medicine, such as cough suppressant, and as a component of skin care products and Japanese drinks, such as batabata-cha [5, 6]. In recent years, the bioactive efficacy and chemical composition of tea flowers have attracted much attention [4, 7]. Polyphenols, saponins, proteins, polysaccharides, and vitamins identified from tea flowers exert biological activities for pharmacological action. Furthermore, the metabolome and transcriptome analyses provide insights into the biosynthesis mechanism of these chemical components [8–10]. Tea flower color and aroma, which are related to pigment distribution type and volatile metabolites, are important features of food garnish. The biosynthesis and relationship between anthocyanins and volatile benzenoid–phenylpropanoids were examined to clarify the regulatory mechanism of color and aroma in the tea flower [8, 10].

The antimicrobial activity of tea leaves has drawn much attention. Studies on tea extracts against different pathogenic microbes have been directed toward inhibiting *Candida* spp., *Listeria monocytogenes*, *Pseudomonas aeruginosa*, *Bacillus cereus*, *Staphylococcus aureus*, *Escherichia coli*, *Streptococcus mutans*, *Lactobacillus acidophilus*, and *Klebsiella pneumoniae* [11–14]. The antimicrobial activity of tea is mainly derived from epigallocatechin gallate (EGCG), epigallocatechin (EGC), epicatechin gallate (ECG), catechins, and caffeine [15–17]. According to the metabolomics and proteomics analysis of *Streptococcus suis* treated with EGCG, several proteins involved in the cell wall, cell membrane, DNA replication, and virulence were downregulated [18]. Meanwhile, RNA-seq analysis revealed that EGCG conferred damages to DNA and envelope and led to iron limitation and oxidative stress in *Pseudomonas fluorescens* [19].

Most studies of the antifungal activities of tea leaves mainly focus on the yeast *Candida*. Limited information has been published concerning the inhibition of filamentous fungi by the tea flower extract (TFE). The antifungal activity of 2-ketobutyric acid in TFE has also been rarely investigated. Aflatoxins produced by *Aspergillus flavus* and *Aspergillus parasiticus* are mycotoxins that can contaminate various foods and agricultural products. Thus, the growth of these fungi and the production of aflatoxin should be inhibited. In the present study, the tea flower collected from Fujian, China was extracted by aqueous liquid and used to investigate free radical scavenging ability, tyrosinase inhibition, and antifungal ability. The chemical composition of tea flower extract was analyzed by total phenols, flavonoids, reducing sugars, and LC-MS. Antimicrobial compounds such as catechin, caffeine, epigallocatechin gallate, salicylic acid, scopoletin, and pyroglutamic acid were identified. The antifungal mechanism of 2-ketobutyric acid against *A. flavus* CCTCC AF 2023038 was also explored by RNA-seq analysis.

## Methods

### Source and extraction of tea (*C. sinensis* L.) flower

Tea flowers were collected from Wuyishan City, Fujian, China, in November 2021, and authenticated by Research Fellow Rongbing Chen (Fujian Academy of Agricultural Sciences). The samples were naturally dried and ground into powder (Fig. S1). The powdered tea flower (100 g) was soaked in deionized water (500 g) at room temperature overnight and sonicated by a JY92-IIN sonicator (Ningbo Scientz Biotechnology, Ningbo, China). The supernatant was filtered and concentrated by reduced pressure and lyophilization to yield a crude extract, namely, TFE (5.5275 g).

### Antioxidant ability of TFE

Radical scavenging assays for 2,2-diphenyl-1-picrylhydrazyl (DPPH) and 2, 2’-azino-bis(3-ethylbenzothiazoline-6-sulfonic acid)(ABTS) were utilized to estimate the antioxidant ability of TFE (0.4, 0.2, 0.1, and 0.05 mg/mL). TFE was mixed with 0.2 mmol/L DPPH solution in darkness at room temperature for 10 min. DPPH radical scavenging was determined by a microplate reader (Molecular Devices, Sunnyvale, CA) at 517 nm. Distilled water was used as control to calculate the DPPH radical scavenging ability (%) of TFE.

ABTS radical scavenging was measured by T-AOC Assay Kit (ABTS) (Beyotime Biotechnology, Shanghai, China). According to the operation manual kit, TFE was added to the ABTS working solution in darkness at room temperature for 5 min. ABTS radical scavenging activity was determined by a microplate reader at 734 nm. Distilled water was used as control to calculate the ABTS radical scavenging ability (%) of TFE.

Ferric-reducing ability of plasma (FRAP) was determined by the T-AOC Assay Kit (FRAP) (Beyotime Biotechnology). According to the operation manual kit, TFE was added into the FRAP working solution at 37℃ for 5 min. If TFE reduces ferric-pyridyltriazine (Fe^3+^- TPTZ), then blue Fe^2+^- TPTZ can be detected by a microplate reader at 593 nm. Different concentrations of Trolox (0.6, 0.3, 0.15, 0.075, and 0.0375 mmol/L) were used as positive control to obtain the standard curve. The antioxidant activity of TFE corresponding to Trolox was determined.

### Nitric oxide scavenging ability of TFE

Nitric oxide (NO) scavenging ability was determined to evaluate the anti-inflammatory ability. Different concentrations of TFE (4, 2, 1, and 0.5 mg/mL) were added to sodium nitroprusside (20 mmol/L) at 25°C for 150 min. The remaining NO in the samples was detected by the Griess reagent (Beyotime Biotechnology) using a microplate reader at 540 nm. Distilled water was used as the control for calculating the NO radical scavenging ability (%) of TFE.

### Inhibition of tyrosinase activity by TFE

The inhibition of tyrosinase activity by TFE was determined using mushroom tyrosinase (Yuanye Bio-Technology, Shanghai, China). Different concentrations of TFE (4, 2, 1, and 0.5 mg/mL) were added to 5 mmol/L dihydroxyphenylalanine (DOPA) (Sangon Biotech, Shanghai, China) and 400 U/mL mushroom tyrosinase at room temperature for 30 min. Distilled water was used as control. The inhibition rate of tyrosinase activity was measured and calculated by a microplate reader at 475 nm.

### Antifungal ability of the TFE

*A. flavus* CCTCC AF 2023038 deposited in the China Center for Type Culture Collection (Hubei, China) was utilized to estimate the antifungal rate of TFE. *A. flavus* spores were scraped from potato dextrose agar in Petri dishes and added into the potato dextrose broth (PDB) with OD600 around 0.1–0.2. Different concentrations of TFE (6.4, 3.2, 1.6, 0.8, and 0.4 mg/mL) were mixed with 1 mL of *A. flavus* broth and added into a 24-well plate at 30℃ for 48 h of incubation. Distilled water was used as control. Fungal growth rate was detected by a microplate reader at 600 nm.

Different concentrations of TFE (6.4, 1.6, and 0.4 mg/mL) were added to 50 mL of *A. flavus* broth with OD600 around 0.1–0.2 and incubated at 30℃ for 48 h. Distilled water was used as control. After 24 and 48 h of cultivation, the supernatant was diluted 50 times for electrical conductivity assay by a conductivity meter (INESA Scientific Instrument, Shanghai, China).

### Total phenol, flavonoid, and reducing sugar contents of the TFE

The total phenol of TFE was determined by Folin–Ciocalteu method. Different concentrations of TFE were mixed with Folin–Ciocalteu reagent for 5 min of reaction. The mixture was added with sodium carbonate solution (10%) in darkness for 30 min and centrifugated at 10,000 ×g (Hettich, Mikro 220R, Germany) for 10 min. The supernatant was collected and measured by a microplate reader at 735 nm. Different concentrations of gallic acid as a positive control were used to prepare the standard curve.

The flavonoid content of TFE was determined by AlCl_3_ method. TFE (0.5 mL) was mixed with methanol (1.5 mL), 10% aluminum chloride (0.1 mL), 1 M potassium acetate (0.1 mL), and distilled water (2.8 mL) at room temperature for 30 min. The supernatant was measured by a microplate reader at 415 nm. Different concentrations of rutin as a positive control were utilized to obtain the standard curve.

The reducing sugar content of TFE was determined by 3,5-dinitrosalicylic acid (DNS) method. Different concentrations of TFE were mixed with DNS solution and heated at 100℃ for 5 min. The supernatant was measured by a microplate reader at 540 nm. Different concentrations of glucose as a positive control were utilized for calculating the standard curve.

### Chemical compounds assay of the TFE by LC-MS

In brief, TFE was dissolved in methanol with 2-amino-3-(2-chloro-phenyl)-propionic acid (4 µg/mL). The sample was ground for 90 s and sonicated for 15 min. The supernatant was obtained by centrifugation and filtered for LC-MS assay. The Vanquish UHPLC System (Thermo Fisher Scientific, USA) fitted with an ACQUITY UPLC ® HSS T3 (150 mm × 2.1 mm, 1.8 μm) (Waters, Milford, MA, USA) was used. For LC-ESI (+)-MS analysis, the mobile phase included (A) 0.1% formic acid in acetonitrile and (B) 0.1% formic acid in water. The condition of the mobile phase was set under the following gradient: 0–1 min (2% A); 1–9 min (2–50% A); 9–12 min (50–98% A); 12–13.5 min (98% A); 13.5–14 min (98–2% A); and 14–20 min (2% A) at 0.25 mL/min. For LC-ESI (-)-MS analysis, the mobile phase included (C) acetonitrile and (D) ammonium formate (5 mmol/L). The condition of the mobile phases was set under the following gradient: 0–1 min (2% C); 1–9 min (2–50% C); 9–12 min (50–98% C); 12–13.5 min (98% C); 13.5–14 min (98–2% C); and 14–17 min (2% C) at 0.25 mL/min. A Q Exactive Focus (Thermo Fisher Scientific) with ESI ion was utilized for mass spectrum detection.

MS1 and MS/MS (Full MS-ddMS2 mode, data-dependent MS/MS) acquisition was conducted with 3.50 and − 2.50 kV for positive and negative modes, respectively. The parameters were as follows: MS1 range of m/z 100–1000 and MS1 resolving power of 70,000 FWHM. Moreover, MS/MS analysis was conducted with resolving power with 17,500 FWHM number by normalized collision energy of 30 eV. Unnecessary MS/MS information was eliminated by dynamic exclusion. BioDeepDB, MoNA, GNPS, mzCloud, and corresponding database (built by Azenta Life Sciences, Suzhou, China) were used to identify metabolites.

### Antifungal ability of 2-keto-glutaramic acid and 2-ketobutyric acid

Fungal antagonistic dosage assay was performed by disc dilution. In brief, 6 mm paper discs with 2-keto-glutaramic acid (0, 2, 4, and 8 mg) and 2-ketobutyric acid (0, 1, 2, and 4 mg) were arranged on a plate with *A. flavus*. The plates were cultivated at 30 °C for 48 h, and a clear zone of antifungal activity was observed.

Different concentrations of 2-keto-glutaramic acid and 2-ketobutyric acid (4, 2, 1, 0.5, 0.25, and 0 mg/mL) were introduced into the *A. flavus* broth with OD600 of 0.1–0.2 for 1 mL. The broth in a 24-well plate was cultivated at 30℃ for 48 h and measured by a microplate reader at 600 nm.

### Propidium iodide staining

*A. flavus* spores were diluted by PDB broth with OD600 of 0.1–0.2. Different concentrations of 2-ketobutyric acid (0, 1, and 2 mg/mL) were introduced into the broth and incubated at 30℃ for 48 h. Fungi were harvested, and PBS buffer was used to wash the hyphae three times. The fungi were suspended in PBS buffer and added with propidium iodide (PI, 100 µg/mL) in darkness for 5 min. Fluorescence images were obtained using a fluorescence microscope (Olympus BX43F-R, Tokyo, Japan).

### RNA-seq analysis of *A. flavus* CCTCC AF 2023038

*A. flavus* spores were added into fresh PDB with OD600 of 0.1–0.2 with and without 2-ketobutyric acid (1 mg/mL). The *A. flavus* broth was cultivated at 30℃ for 48 h. *A. flavus* was harvested and sent to Majorbio Bio-pharm Technology Co., Ltd. (Shanghai, China) for RNA extraction, RNA-seq, and bioinformatic analysis. cDNA sequencing was performed on an Illumina NovaSeq 6000 (Illumina, San Diego, CA). The sequencing reads were assembled, evaluated, and aligned by Trinity [20], BUSCO [21], and HMMER3 [22]. The unigenes were annotated by NCBI_NR, GO, and KEGG analyses. Quantitative transcriptome analysis was carried out by RSEM [23]. Differentially expressed genes (DEGs) were compared by DESeq2 with the criteria of p-adjust < 0.05 and FC (Fold Change) > 2. These RNA-seq data were deposited at DDBJ/ENA/GenBank under the accession no. of BioProject PRJNA939021 and SRA with SRR23684269, SRR23684270, SRR23684271, SRR23684272, SRR23684273, and SRR23684274.

### Gene expression analysis by real-time RT-PCR

RNA was extracted using TRIzol reagent (Thermo Fisher Scientific) to confirm the result of RNA-seq. First-strand cDNA was synthetized by HiFi-MMLV cDNA kit with oligo (dT) and random hexamers (Beijing ComWin Biotech, China) at 42℃ for 1 h. The UltraSYBR Mixture (Beijing ComWin Biotech) was mixed with cDNA in a final volume of 25 µL. Real-time PCR was conducted on Roche LightCycler® 480 System (Roche Group, Switzerland) under thermal process for 40 cycles including denaturation at 95℃ for 10 s, annealing at 57℃ for 30 s, and extension at 72℃ for 32 s. The primers utilized are shown in Table S1.

### Quantification of aflatoxins

*A. flavus* spores were added to 50 mL of fresh PDB with OD600 of 0.1–0.2 with and without 2-ketobutyric acid (1 mg/mL) at 30℃ for 14 d and 150 rpm under shaking incubation (THZ-98 C, Bluepard Instruments, Shanghai, China). The culture supernatant was harvested, and aflatoxins were detected by HPLC system (LC-20AD, Shimadzu, Tokyo, Japan) with a C18 column (5 μm, 4.6 mm × 150 mm, Shimadzu) and a fluorescence detector (RF-20 A, Shimadzu). The mobile phase comprised 40% methanol and 60% H_2_O, with a 1 mL/min flow rate for 25 min. Aflatoxins (B1, B2, G1, G2, M1, and M2) were purchased from Guanyibio Co., Ltd. (Jiangsu, China) for calibration curve analysis.

### Statistical analysis

Data were reported using mean and standard deviation. Duncan’s multiple range test at a confidence level of 95% was performed using IBM SPSS Statistics v20 software package (SPSS Inc. Chicago, USA).

## Results

### Effect of TFE on the antioxidant ability

**Fig. 1:**
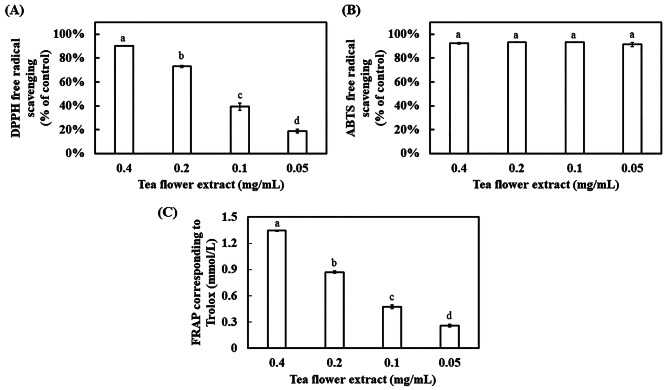
Antioxidant activity of TFE. Free radical scavenging ability was determined by the **(A)** DPPH and **(B)** ABTS assays. Sterile distilled water was used as a control. Ferric-reducing ability was detected by the T-AOC Assay Kit (FRAP). Different concentrations of **(C)** Trolox were used as a positive control. Results are presented as mean ± S.D. (*n* = 3)

The antioxidant activity of TFE was estimated by DPPH and ABTS free radical scavenging assays. The amount of DPPH free radicals eliminated increased with increasing TFE concentration (Fig. 1A). The DPPH free radical scavenging reached 90% when 0.4 mg/mL TFE was used, corresponding to that of 20 µg/mL ascorbic acid (Fig. S2). Moreover, TFE removed ABTS free radicals very well. The ABTS free radical scavenging activity reached more than 90% when TFE lower than 0.05 mg/mL was used (Fig. 1B), corresponding to that of 0.3 mmol/L Trolox (Fig. S2).

FRAP assay was used to evaluate whether TFE can reduce Fe^3+^-TPTZ to produce blue Fe^2+^- TPTZ to evaluate antioxidant capacity. The reducing ability of Fe^3+^- TPTZ tended to increase with increasing TFE concentration. The effect of 0.1 mg/mL TFE corresponded to that of 0.48 mmol/L Trolox (Fig. 1C).

### Effect of TFE on the anti-inflammatory ability and the inhibition of tyrosinase activity

**Fig. 2:**
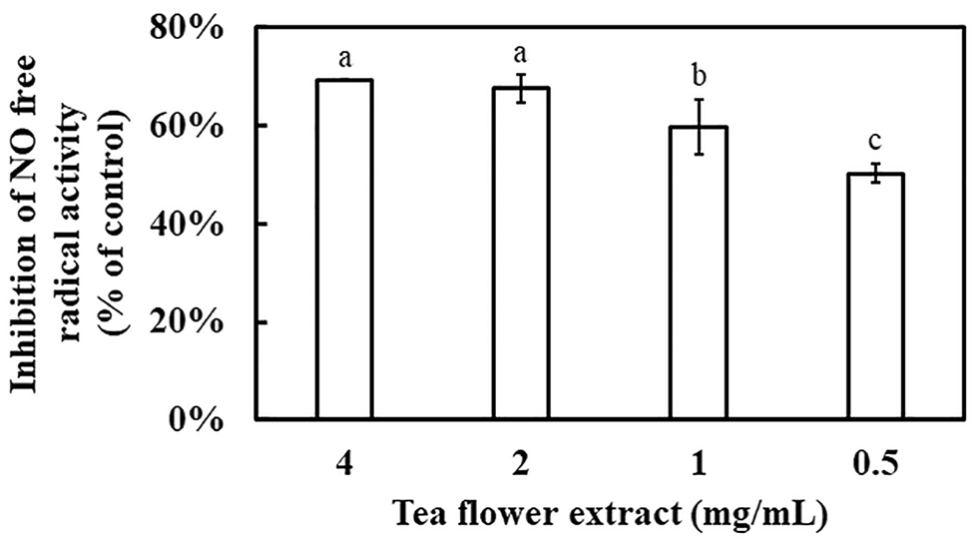
Determination of NO free radical scavenging ability by TFE. Results are presented as mean ± S.D. (*n* = 3)

The increase in NO content is associated with inflammation, cancer, and various human diseases [24]. Therefore, NO free radical scavenging was evaluated using different concentrations of TFE. The inhibition of NO free radicals increased with increasing TFE concentration (Fig. 2). Nevertheless, the dose of TFE in inhibiting the NO free radical was not as good as that of DPPH and ABTS free radicals.

A previous study indicated that the tea flower extract can prevent the biosynthesis of melanin in the B16-F10 cell [25]. In the present study, tyrosinase activity, which is involved in melanin production, was used to estimate the capacity of melanin inhibition by TFE. Tyrosinase activity was not inhibited when 4 mg/mL TFE was used.

### Effect of TFE on the antifungal activity

**Fig. 3:**
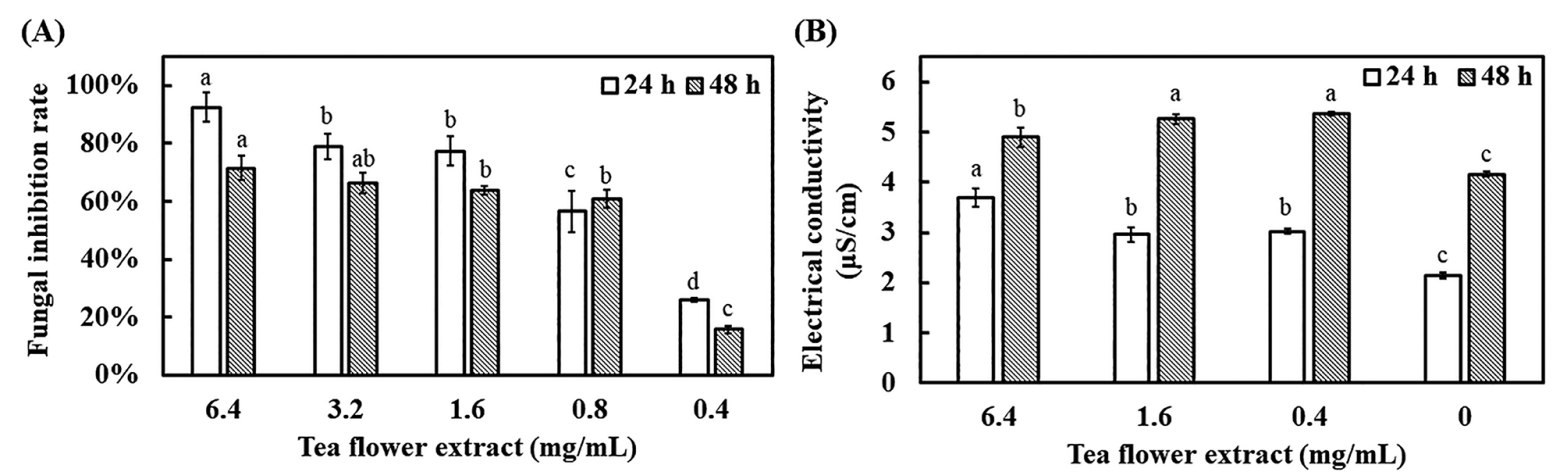
Antifungal activity of TFE. **(A)** *A. flavus* CCTCC AF 2023038 broth (1 mL) treated with different concentrations of TFE was introduced into a 24-well plate and cultivated at 30°C for 24 and 48 h. The broth was analyzed by a microplate reader at 600 nm. **(B)** About 50 mL of *A. flavus* CCTCC AF 2023038 broth treated with different concentrations of TFE was used for electrical conductivity assay. Results are presented as mean ± S.D. (*n* = 3)

*A. flavus* was utilized to evaluate the antifungal activity of TFE. The result revealed that TFE significantly retarded fungal growth (Fig. 3A). The fungal inhibition rate reached more than 90% after 24 h of incubation with 6.4 mg/mL TFE. When the TFE concentration was decreased to 0.4 mg/mL, the fungal inhibition rate was maintained at 25.9%. In addition, the antifungal activity was slightly reduced only after 48 h of incubation. Overall, 6.4 mg/mL TFE had 71.5% antifungal rate, whereas 0.8 mg/mL TFE demonstrated 60% inhibition rate after 48 h of incubation.

When the cell membrane is damaged, soluble substances will be released, resulting in an increase in electrical conductivity [26]. The electrical conductivity of the broth with and without TFE was determined to verify whether TFE will damage the cell membrane. After 24 h incubation of *A. flavus* treated with TFE, the electrical conductivity increased with increasing TFE concentration, indicating its harmful effect on the fungus (Fig. 3B). Similar results were observed after 48 h of incubation. However, the difference in electrical conductivity between 24 and 48 h of incubation decreased when 6.4 mg/mL TFE was used. Given that the growth of *A. flavus* was prohibited, the leakage of soluble substances was reduced.

### Total phenol, flavonoid, and reducing sugar assays of TFE

Various polyphenols, flavonoids, and polysaccharides had been reported to contribute to different biological abilities [27]. Thus, Folin-Ciocalteu, AlCl_3_, and DNS methods in the present study were utilized to assay the TFE. The result showed that the TFE had 26.6 ± 1.0, 20.0 ± 4.1, and 218 ± 7.2 mg/g corresponding to gallic acid, rutin, and glucose, respectively.

### Chemical compound assay of TFE by LC-MS

Metabolites derived from TFE were identified through LC-MS analysis. Table 1 shows the 24 compounds with the highest contents in TFE. The characteristic fragmentation patterns of compounds with mass-to-charge ratio (m/z) were verified and compared by the metabolite database (Fig. S3). According to the classification of compounds, amino acids, peptides, and analogs including L-theanine, L-phenylalanine, L-isoleucine, L-glutamic acid, 5-aminopentanoic acid, pyroglutamic acid, and L-tyrosine were the most abundant, following by carbohydrates and carbohydrate conjugates including D-galactose, D-fructose, D-xylose, threonic acid, and muramic acid. The metabolites with the highest contents were D-galactose, followed by L-theanine, catechin, and caffeine.

**Table 1:** The most abundant compounds of the TFE identified by LC-MS.

Compounds	Subclass	mz	Retention time (s)	Mode	Relative abundance	Relative content (%)
D-Galactose	Carbohydrates and carbohydrate conjugates	179.056	91.8	neg	3.116E + 10 ± 6.95E + 08	15.27%
L-Theanine	Amino acids, peptides, and analogues	175.108	109.7	pos	2.192E + 10 ± 4.55E + 08	10.75%
Catechin	Flavans	289.071	414.1	neg	2.179E + 10 ± 3.24E + 09	10.68%
Caffeine	Purines and purine derivatives	195.086	438.5	pos	1.022E + 10 ± 9.3E + 09	5.01%
L-Phenylalanine	Amino acids, peptides, and analogues	166.085	302.4	pos	8.245E + 09 ± 3.78E + 08	4.04%
L-Isoleucine	Amino acids, peptides, and analogues	132.102	191.2	pos	6.873E + 09 ± 2.56E + 08	3.37%
Astragalin	Flavonoid glycosides	447.093	491.9	neg	5.245E + 09 ± 1.8E + 09	2.57%
D-Fructose	Carbohydrates and carbohydrate conjugates	179.056	103.2	neg	4.036E + 09 ± 3.71E + 09	1.98%
Scopoletin	Hydroxycoumarins	191.035	575	neg	4.377E + 09 ± 8.7E + 07	2.15%
L-Glutamic acid	Amino acids, peptides, and analogues	130.049	156.6	pos	3.53E + 09 ± 1.5E + 08	1.73%
4-Hydroxycinnamoylagmatine	Hydroxycinnamic acids and derivatives	276.144	243.6	pos	3.337E + 09 ± 7.5E + 08	1.64%
D-Xylose	Carbohydrates and carbohydrate conjugates	149.044	89.9	neg	3.328E + 09 ± 1.1E + 08	1.63%
5-Aminopentanoic acid	Amino acids, peptides, and analogues	118.085	104.5	pos	3.162E + 09 ± 9.7E + 07	1.55%
Epigallocatechin gallate	Flavans	457.077	416.7	neg	3.059E + 09 ± 2.2E + 08	1.50%
Salicylic acid	Benzoic acids and derivatives	137.023	350.5	neg	2.865E + 09 ± 5.4E + 07	1.40%
2-Keto-glutaramic acid	Short-chain keto acids and derivatives	145.048	94.3	pos	2.652E + 09 ± 6.1E + 0.8	1.30%
Adenine	Purines and purine derivatives	134.046	246	neg	2.502E + 09 ± 6E + 07	1.23%
Threonic acid	Carbohydrates and carbohydrate conjugates	135.029	82.4	neg	2.417E + 09 ± 9E + 07	1.18%
3-Hydroxymethylglutaric acid	Fatty acids and conjugates	145.049	283.6	pos	2.339E + 09 ± 3.5E + 08	1.15%
(S)-2-Propylpiperidine	-	128.143	836.8	pos	2.224E + 09 ± 1.1E + 0.8	1.09%
Muramic acid	Carbohydrates and carbohydrate conjugates	234.097	106.3	pos	2.11E + 09 ± 7.7E + 07	1.03%
Pyroglutamic acid	Amino acids, peptides, and analogues	128.036	89.7	neg	2.051E + 09 ± 1.1E + 0.8	1.01%
L-Tyrosine	Amino acids, peptides, and analogues	182.081	185.8	pos	1.979E + 09 ± 3.9E + 07	0.97%
2-Ketobutyric acid	Short-chain keto acids and derivatives	101.024	91.8	neg	1.828E + 09 ± 9.7E + 07	0.90%

### Effect of the different metabolites on the antifungal activity

**Fig. 4:**
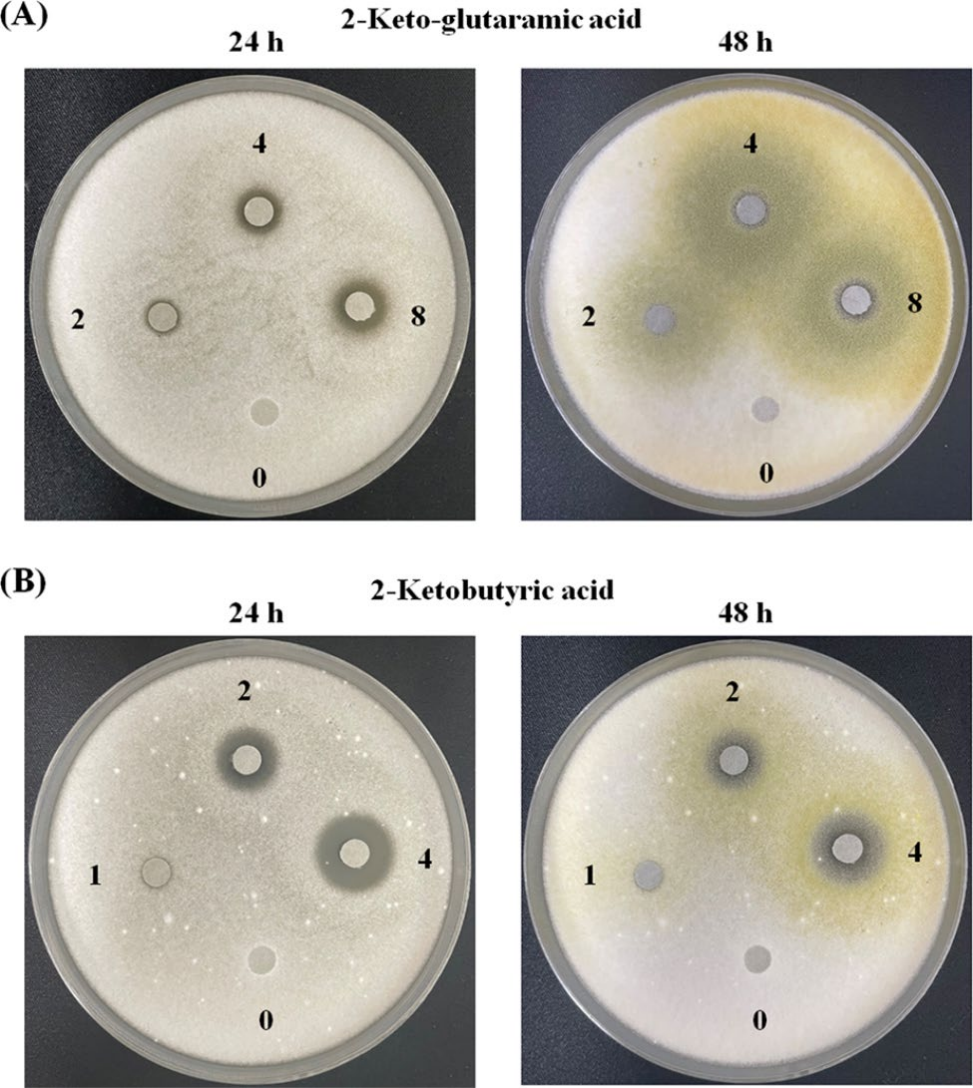
Antifungal activity of **(A)** 2-keto-glutaramic acid and **(B)** 2-ketobutyric acid. Paper discs with 2-keto-glutaramic acid (0, 2, 4, and 8 mg) and 2-ketobutyric acid (0, 1, 2, and 4 mg) were placed on plates with spread *A. flavus* CCTCC AF 2023038. The plates were cultured at 30°C for 24 and 48 h

Catechin, caffeine, epigallocatechin gallate, salicylic acid, scopoletin, and pyroglutamic acid, which were identified in TFE, have antimicrobial capacity [28–32]. Therefore, other compounds including L-phenylalanine, L-isoleucine, astragalin, 2-keto-glutaramic acid, L-tyrosine, and 2-ketobutyric acid were analyzed to confirm whether they can inhibit *A. flavus*. A fungal antagonistic dosage assay was performed to explore the activity of these compounds by disc diffusion. However, only 2-keto-glutaramic acid and 2-ketobutyric acid had an evident clear zone (Fig. 4). The clear zone of 2-ketobutyric acid was more obvious than that of 2-keto-glutaramic acid. Moreover, 2-ketobutyric acid had more visible inhibition at 2 mg than 2-keto-glutaramic acid at 8 mg after 24 h of incubation. Nevertheless, the clear zones were reduced after 48 h of cultivation in both compounds.

**Fig. 5:**
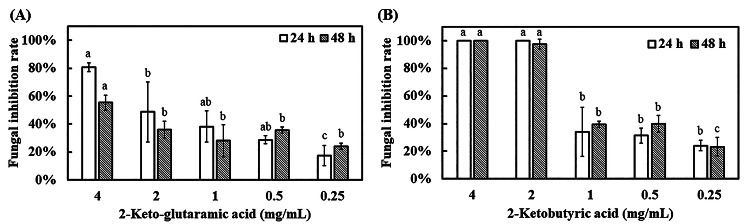
Antifungal activity of **(A)** 2-keto-glutaramic acid and **(B)** 2-ketobutyric acid. *A. flavus* CCTCC AF 2023038 broth (1 mL) treated with different concentrations of 2-keto-glutaramic acid and 2-ketobutyric acid was introduced into a 24-well plate and cultivated at 30°C for 24 and 48 h. The broth was analyzed by a microplate reader at 600 nm. Results are presented as mean ± S.D. (*n* = 3)

*A. flavus* broth treated with 2-keto-glutaramic acid and 2-ketobutyric acid was analyzed at OD600 after 48 h of incubation to verify the antifungal rate. The result showed that 2 mg/mL 2-keto-glutaramic acid had only half of the fungal inhibition rate after 24 h of incubation (Fig. 5). By contrast, 2 mg/mL 2-ketobutyric acid achieved more than 90% antifungal rate after incubation for 24 and 48 h. According to calculation by GraphPad Prism 9 (GraphPad Software, MA, USA), the IC50 values of 2-keto-glutaramic acid and 2-ketobutyric acid after 48 h of cultivation were 2.136 and 1.232 mg/mL, respectively. Hence, *A. flavus* was more sensitive to 2-ketobutyric acid than to 2-keto-glutaramic acid, consistent with the results of the disc diffusion assay.

### Effect of 2-ketobutyric acid on the cell membrane integrity of *A. flavus* CCTCC AF 2023038

**Fig. 6:**
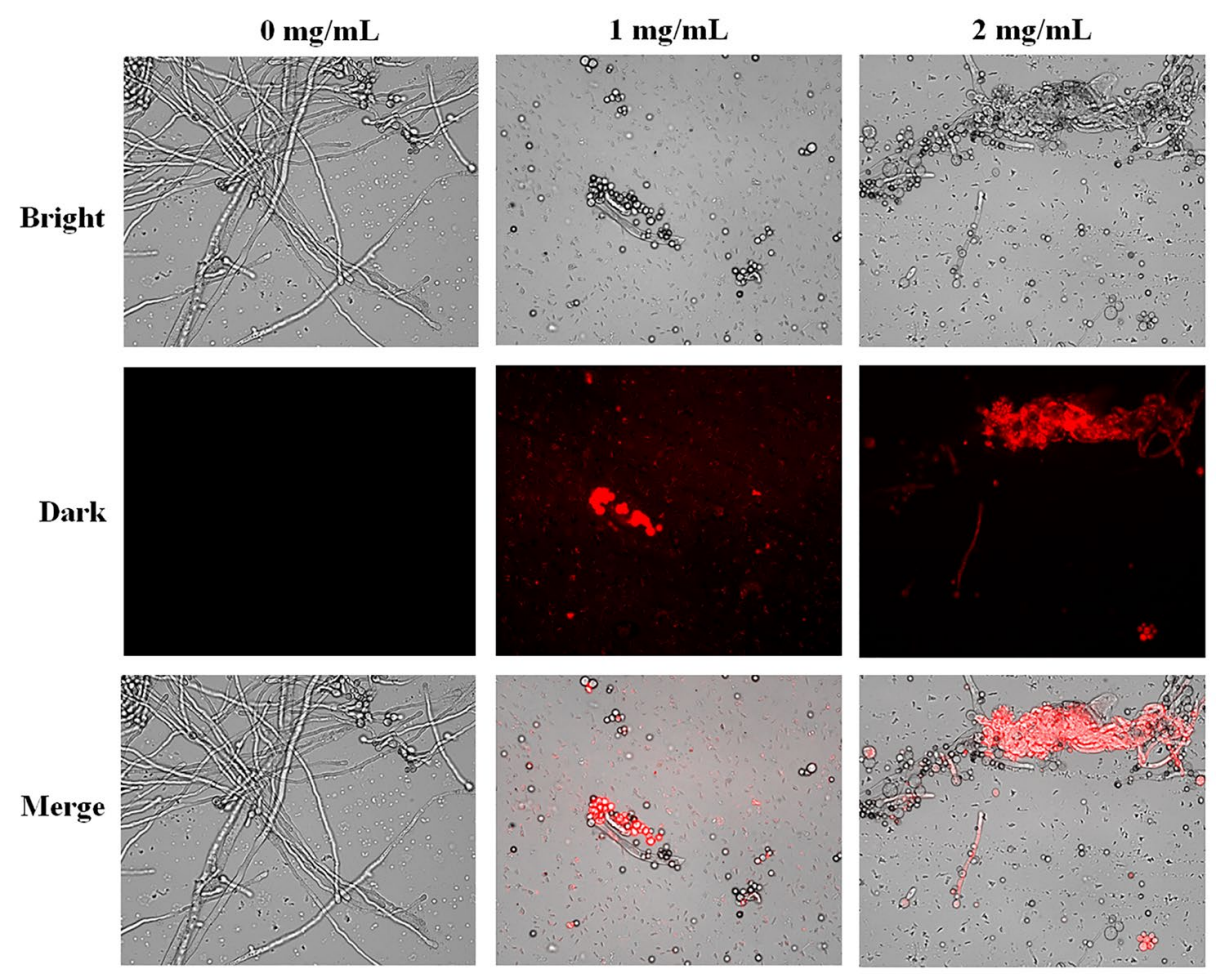
* A. flavus* CCTCC AF 2023038 treated with 2-ketobutyric acid (0, 1, and 2 mg/mL). Morphology was observed under bright field and the corresponding PI staining under dark field

Considering that 2-ketobutyric acid had better antifungal ability than 2-keto-glutaramic acid, we examined its antifungal mechanism by PI staining and RNA-seq analysis. A fluorescent dye for PI staining was used to verify whether 2-ketobutyric acid will influence the integrity of the cell membrane. A red fluorescence can be observed if the cell membrane is damaged, and the PI dye will enter the cell to react with DNA and RNA. In this study, no red fluorescence was observed in the control without 2-ketobutyric acid, suggesting the intact cell membrane of *A. flavus*. Nevertheless, red fluorescence was detected in *A. flavus* treated with 1 and 2 mg/mL 2-ketobutyric acid (Fig. 6). Moreover, the fluorescence intensity increased with increasing amount of 2-ketobutyric acid, indicating that it damaged the fungal cell membranes.

### RNA-seq analysis of *A. flavus* CCTCC AF 2023038 with 2-ketobutyric acid treatment

**Fig. 7:**
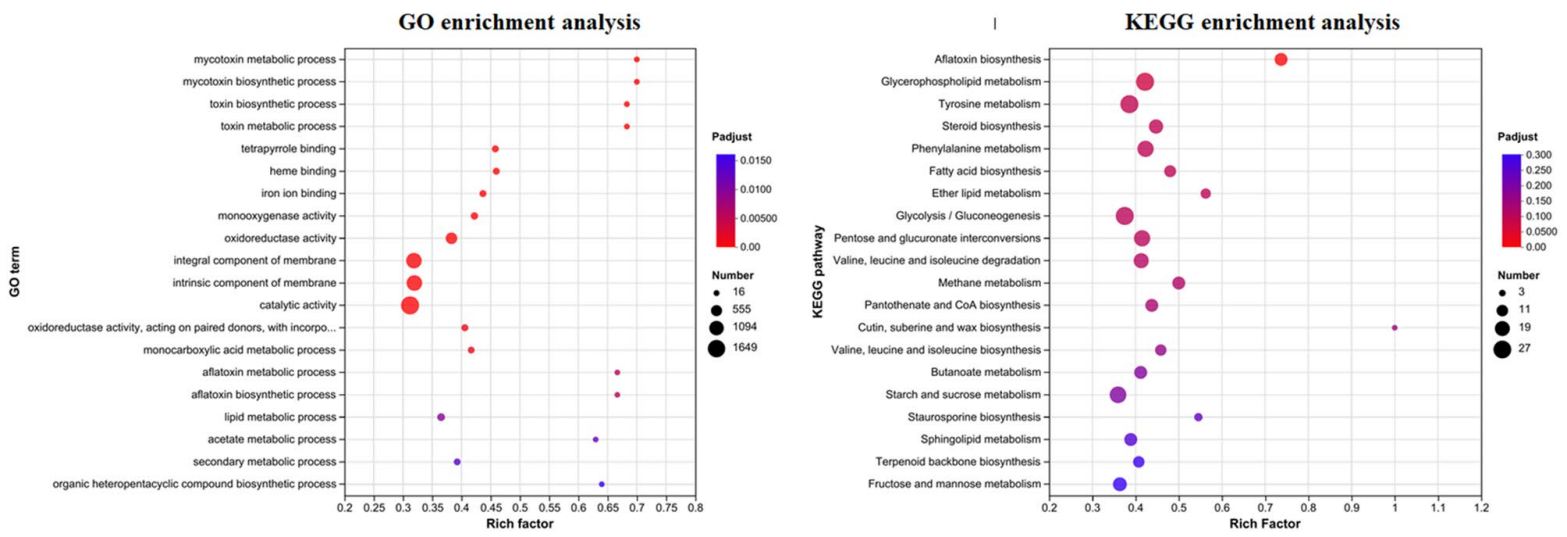
Classification of DEGs by *A. flavus* CCTCC AF 2023038 treated with and without 2-ketobutyric acid according to the GO and KEGG enrichment analyses

*A. flavus* treated with and without 2-ketobutyric acid (1 mg/mL) was subjected to transcriptome analysis. Over 56 Gb of raw bases and an average of 61,417,767 clean reads were obtained from each treatment in triplicate (Table S2). After assembling the clean reads of each sample, the total number of unigenes was 14,618, which were annotated for 9,662, 4,365, and 12,549 functional unigenes by the GO, KEGG, and NCBI_NR databases, respectively. The triplicate of each sample with and without 2-ketobutyric acid reached a correlation with an average of 0.95 by the Pearson correlation analysis (Fig. S4). DEGs in *A. flavus* after 2-ketobutyric acid treatment accounted for 3,808 genes as fold change of more than 2 by volcano plot analysis. About 1,298 upregulated and 2,510 downregulated genes were recorded. The 20 most upregulated and downregulated genes by 2-ketobutyric acid treatment are presented in Tables S3 and S4. In addition, 3808 and 1,051 DEGs were recognized by the GO and KEGG databases (Fig. S5). In the GO database, the most DEGs involved in response to catalytic activity, membrane part, and metabolic process were identified in Molecular Function, Cellular Component, and Biological Process. On the other hand, the most DEGs involved in response to carbohydrate metabolism, transport and catabolism, folding, sorting and degradation, signal transduction, and aging were shown in Metabolism, Cellular Processes, Genetic Information Processing, Environmental Information Processing, and Organismal Systems based on the KEGG database [33–35]. According to the GO and KEGG enrichment analyses, a high rich factor and a low p-adjust value were obtained in the mycotoxin metabolic process, mycotoxin biosynthetic process, and aflatoxin biosynthesis (Fig. 7). Moreover, the multidrug transporter genes associated with the resistance of fungicides were affected. Thus, the effect of 2-ketobutyric acid on the genes involved in the multidrug transporter was summarized (Table 2).

**Table 2:** The multidrug resistance transporter genes responding to the ratio of the 2-ketobutyric acid treatment to control in *A. flavus* CCTCC AF 2023038

No.	Gene ID	NR description	FC	Log_2_FC	P-value	Regulation
1	TRINITY_DN3612_c0_g1	putative MFS multidrug transporter	17.88	4.160308	2.89E-38	Up
2	TRINITY_DN1582_c0_g1	MFS multidrug transporter	3.338	1.738917	1.40E-10	
3	TRINITY_DN2880_c0_g1	putative MFS multidrug transporter	2.622	1.390627	1.92E-07	
4	TRINITY_DN3169_c0_g1	MFS multidrug transporter	2.286	1.192875	3.90E-08	
5	TRINITY_DN904_c0_g1	MFS multidrug transporter	2.185	1.127846	1.61E-05	
6	TRINITY_DN2191_c0_g1	MFS transporter	2.067	1.047429	0.004091	
7	TRINITY_DN5972_c0_g2	ABC multidrug transporter	0.021	-5.5459	0.00059	Down
8	TRINITY_DN10177_c0_g1	major facilitator superfamily MFS_1	0.024	-5.36172	8.34E-29	
9	TRINITY_DN11756_c0_g1	MFS multidrug transporter	0.041	-4.60702	2.04E-05	
10	TRINITY_DN1139_c0_g1	multidrug resistance-associated protein	0.042	-4.56916	7.81E-79	
11	TRINITY_DN9541_c0_g1	MFS general substrate transporter	0.043	-4.52672	0.02107	
12	TRINITY_DN9177_c0_g1	ABC multidrug transporter	0.048	-4.36709	0.008804	
13	TRINITY_DN9432_c0_g1	MFS transporter	0.054	-4.21978	1.02E-15	
14	TRINITY_DN4236_c0_g1	MFS transporter	0.054	-4.21605	7.27E-28	
15	TRINITY_DN11504_c0_g1	multidrug resistance protein CDR1	0.074	-3.7552	3.14E-50	
16	TRINITY_DN147_c0_g1	MFS transporter	0.11	-3.17817	9.78E-54	
17	TRINITY_DN1363_c0_g1	multidrug resistance-associated protein	0.151	-2.72974	5.91E-28	
18	TRINITY_DN2335_c0_g1	major facilitator superfamily	0.195	-2.35749	6.52E-11	
19	TRINITY_DN2570_c0_g1	putative MFS transporter	0.218	-2.19513	1.13E-24	
20	TRINITY_DN5232_c1_g1	MFS transporter	0.247	-2.01706	1.45E-18	
21	TRINITY_DN4911_c0_g1	MFS monocarboxylate transporter	0.283	-1.82286	4.04E-08	
22	TRINITY_DN6896_c0_g2	putative MFS transporter	0.291	-1.77968	2.62E-06	
23	TRINITY_DN1255_c0_g1	multidrug resistance-associated protein	0.309	-1.69607	1.30E-19	
24	TRINITY_DN10251_c0_g1	MFS multidrug transporter	0.311	-1.68635	0.001655	
25	TRINITY_DN6368_c0_g4	MFS multidrug transporter	0.335	-1.57677	0.000324	
26	TRINITY_DN3325_c0_g1	major facilitator superfamily MFS_1	0.388	-1.36645	2.83E-14	
27	TRINITY_DN3043_c0_g1	MFS transporter	0.434	-1.20475	9.17E-05	
28	TRINITY_DN2439_c0_g1	MFS transporter	0.479	-1.06074	0.000658	
29	TRINITY_DN4691_c0_g1	MFS drug transporter	0.492	-1.02402	7.81E-05	

Six genes containing three upregulated genes and three downregulated genes were randomly selected for real-time RT-PCR analysis to confirm the RNA-seq result. The gene regulation data using real-time RT-PCR were in agreement with the change in RNA-seq with and without 2-ketobutyric acid treatment (Table S5).

### Aflatoxin analysis of *A. flavus* CCTCC AF 2023038 with 2-ketobutyric acid treatment

**Fig. 8:**
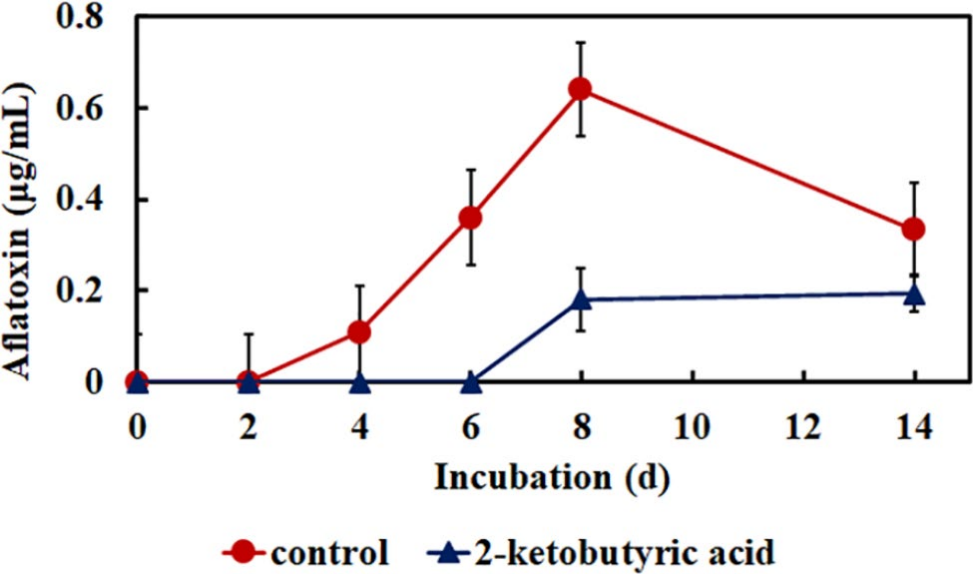
Aflatoxin production of *A. flavus* CCTCC AF 2023038 treated with and without 2-ketobutyric acid after 14 d of incubation. Results are presented as mean ± S.D. (*n* = 3)

The time course of aflatoxin was analyzed after 14 d of incubation to determine the effect of 2-ketobutyric acid on the aflatoxin production of *A. flavus*. Aflatoxin G1 was detected on the fourth and 8th day in the absence and presence of 1 mg/mL 2-ketobutyric acid, respectively (Fig. 8). The aflatoxin G1 production in the presence of 2-ketobutyric acid was lower than that of the control. After 14 d of incubation, the mycelia were harvested and the amounts of aflatoxin G1 in the control and 2-ketobutyric acid treatment were 86.2 and 49.3 µg/g, respectively.

## Discussion

Tea is usually brewed with an aqueous liquid, so water was used to extract tea flower in this study. The result of reducing sugar assay by DNS is consistent with the chemical compound assay by LC-MS, revealing the highest content of carbohydrates in TFE. In particular, D-galactose, D-fructose, and D-xylose were the most abundant compounds in TFE (Table 1). These reducing sugars were formed by the biosynthesis and degradation of polysaccharides, which are the most typical components found in tea [36]. The second most abundant metabolite is L-theanine, which is the most abundant free amino acid in tea as reported in a previous study [37]. Moreover, the second most abundant amino acid is L-phenylalanine, which accumulates in tea flower during floral development [38]. Interestingly, the high content of isoleucine has been rarely reported in the tea flower. In addition, common compounds in tea such as catechin, epigallocatechin gallate, and caffeine, corresponding to flavonoid, and purine alkaloid have been found in TFE, suggesting that tea leaves and flowers have similar components [2]. Among the eight catechins identified in tea flowers, catechin and epigallocatechin gallate were the most abundant in TFE; this is responsible for the existence of epigallocatechin gallate with the highest content at all stages of flowering [36]. Furthermore, a high-yield caffeine (purine alkaloid) responded to a high-yield adenine involved in the biosynthesis of purine alkaloids [39].

TFE exhibited excellent free radical scavenging of DPPH and ABTS as well as ferric-reducing ability, corresponding to antioxidant capacity (Fig. 1). Moreover, NO free radical scavenging activity, corresponding to anti-inflammatory capacity, was found in TFE (Fig. 2). The effect of TFE on anti-oxidation may be contributed by its high contents of L-theanine, catechin, caffeine, astragalin, scopoletin, and epigallocatechin gallate, which belong to amino acids, flavans, purines and purine derivatives, flavonoid glycosides, and hydroxycoumarins [40–44]. Nevertheless, TFE had different effects on the removal of free radicals, following the order of ABTS > DPPH > NO.

The catalysis of tyrosinase is a rate-limiting step in melanin biosynthesis [45]. L-Phenylalanine is catalyzed into L-tyrosine by phenylalanine hydroxylase and is further converted into L-dopaquinone by tyrosinase. L-Dopaquinone is transformed into pheomelanin in the presence of glutathione or cysteine. On the other hand, L-dopaquinone is subsequently catalyzed into eumelanin by tyrosinase, tyrosinase related protein-2 (TRP-2), and tyrosinase related protein-1 (TRP-1). Our result differs from the report of Dissanayake et al., who stated that green tea flower extract (GTFE) can inhibit tyrosinase activity and melanin synthesis in B16-F10 melanoma cells [25]. Tyrosinase activity was not inhibited by TFE, which may be due to its large amount of L-tyrosine catalysts of tyrosinase. Although the known inhibitor, pyroglutamic acid, was present in TFE, its inhibition ability was insufficient to inhibit the catalysis of tyrosinase [46].

Although a number of research has been published on the antibacterial capacity of tea leaf extracts, limited attention has focused on the inhibition of filamentous fungi. *A. flavus* was used as a model of antifungal activity by TFE. The result indicated that TFE had more than half the growth inhibition rate at the centration of 0.8 mg/mL even after 2 days of culture (Fig. 3). In addition, the increase in the electrical conductivity in the fungal broth treated with TFE implied the damages to the cell wall and cell membrane, leading to cytoplasm leakage and fungal death [26]. Several antimicrobial agents were identified, such as catechin, caffeine, epigallocatechin gallate, salicylic acid, scopoletin, and pyroglutamic acid, according to the chemical composition of TFE [28–31]. In the present work, L-phenylalanine, L-isoleucine, astragalin, 2-keto-glutaramic acid, L-tyrosine, and 2-ketobutyric acid were further explored for antifungal activity. Only two compounds, namely, 2-keto-glutaramic acid and 2-ketobutyric acid, had significant inhibitory effects (Fig. 4). To the best of our knowledge, no relevant literature has been reported about the fungal inhibition of the two compounds. Furthermore, the antifungal effect of 2-ketobutyric acid was better than that of 2-keto-glutaramic acid (Fig. 5). 2-Keto-glutaramic acid is widely found in various organisms and can be obtained through glutamine transformation. As such, it could be used a biomarker of hepatic encephalopathy and other hyperammonemic diseases [47, 48]. Meanwhile, 2-ketobutyric acid is usually applied as a food flavoring and serves as a substrate for the synthesis of amino acids, proteins, sugars, fats, and porphyrins [49]. It has a FEMA (Flavour Extract Manufacturers Association) number of 3723 and considered as Generally Recognized as Safe (GRAS). Given that 2-ketobutyric acid is safe to use in the food industry, its antifungal mechanism was further examined by PI staining and RNA-seq analysis.

The result of PI staining with *A. flavus* treated by 2-ketobutyric acid is consistent with that of electrical conductivity, indicating that TFE led to cell membrane damage and permeability imbalance (Fig. 6). The antifungal activity of 2-ketobutyric acid was further evaluated through transcriptome analysis. The damage to cell membranes was reflected in the efflux transporters associated with multidrug resistance (MDR) [50]. Among 29 multidrug resistance transporter genes, 23 genes associated with MFS transporters (major facilitator superfamily transporters) and ABC transporters (ATP binding cassette superfamily transporters) were downregulated (Table 2). The cell membrane, which is composed of sterols, sphingolipids, and glycerophospholipids, is an important channel for material exchange between the cell and the surrounding environment; as such, fungicides mainly target the cell membrane integrity [51]. The well-known fungicides, polyene and azoles, could bind to ergosterol or inhibit its synthesis, causing damages to cell membranes [52]. In the present study, more than 1,000 DEGs in *A. flavus* involved in the integral and intrinsic component of the cell membrane were influenced by 2-ketobutyric acid based on the GO enrichment analysis (Fig. 7). Moreover, several DEGs were observed in steroid biosynthesis (17 DEGs), sphingolipid metabolism (14 DEGs), and glycerophospholipid metabolism (27 DEGs) in the KEGG enrichment analysis, and most of the genes were downregulated. Interestingly, all of the genes involved in sphingolipid metabolism were downregulated. This finding suggests that 2-ketobutyric acid may prevent sphingolipid synthesis and damage the cell membranes. Studies have reported on australifungin, lipoxamycin, and fumonisins as sphingolipid synthesis inhibitors to suppress sphinganine N-acyltransferase, serine palmitoyl-transferase, and ceramide synthase activity [53–55]. For molecular docking analysis, the amino acid of serine palmitoyl-transferase was retrieved from the NCBI database with the accession number PYH86761 and a 3D model was generated by the SWISS-MODEL (https://swissmodel.expasy.org). The affinity of 2-ketobutyric acid to serine palmitoyl-transferase was − 4.4 kcal/mol, while that of lipoxamycin was − 5.1 kcal/mol according to the AutoDock Vina (https://autodock.scripps.edu). Furthermore, two hydrogen bonds of 2-ketobutyric acid were detected with SER292 and MET293 of serine palmitoyl-transferase (Fig. S6). This finding implies that 2-ketobutyric acid could bind to serine palmitoyl-transferase, thereby reducing cell membrane synthesis. Therefore, the antifungal mechanism of 2-ketobutyric acid deserved further investigation.

A highly rich factor associated with mycotoxin metabolic and biosynthetic processes, and aflatoxin biosynthesis was perceived in the GO and KEGG enrichment analyses, respectively (Fig. 7). According to the annotation of NCBI_NR and KEGG pathway, DEGs associated with aflatoxin biosynthesis genes included AflL, AflM, AflP, AflV, and AflX, which were downregulated. This finding indicates that the addition of 2-ketobutyric acid could decrease the production of fungal mycotoxin. The aflatoxin level decreased upon treatment with 2-ketobutyric acid (Fig. 8). Several compounds, such as magnolol, citral, and thymol, can also reduce the mycotoxin production of alternariol (AOH), alternariol monomethyl ether, deoxynivalenol (DON), and 3-Ac-DON and inhibit *Alternaria alternata*, and *Fusarium graminearum* [56–58]. Furthermore, an aflatoxin derived from *Aspergillus* spp. was found to belong to a kind of polyketide that is composed of biopolymers of acetate and short carboxylates [59]. In the present study, acetate metabolism was downregulated in the GO enrichment analysis. Thus, the decline in the levels of mycotoxin and acetate metabolism-related genes was consistent.

## Conclusions

Studies on tea flowers have been devoted to their health benefits, such as antioxidant, anti-inflammatory, immunostimulating, antitumor, hypoglycemic, hypolipidemic, anti-obesity, antiallergic, and gastroprotective effects. Our research indicated the presence of several well-known disinfectants such as catechin, caffeine, epigallocatechin gallate, salicylic acid, scopoletin, and pyroglutamic acid in TFE, suggesting its potential to be an antimicrobial agent. 2-Ketobutyric acid has been widely applied in the food industry, and its antifungal capacity enhanced its application value. According to the PI staining and RNA-seq analyses, 2-ketobutyric acid may inhibit sphingolipid synthesis associated with the component of the cell membrane. The addition of 2-ketobutyric acid downregulates the biosynthesis of mycotoxins, such as aflatoxin, which result in crop contamination. To the best of our knowledge, this study is the first to report the antifungal activity of 2-ketobutyric acid. In the future, fungicides with strong antifungal ability can be synthesized or designed according to the skeleton of 2-ketobutyric acid.

### Electronic supplementary material

Below is the link to the electronic supplementary material.

Supplementary Material 1

Supplementary Material 2

Supplementary Material 3

Supplementary Material 4

Supplementary Material 5

Supplementary Material 6

Supplementary Material 7

Supplementary Material 8

Supplementary Material 9

Supplementary Material 10

Supplementary Material 11

## Data Availability

The datasets generated and analyzed during the current study are available in DDBJ/ENA/GenBank under the accession no. of BioProject PRJNA939021, and SRA with SRR23684269, SRR23684270, SRR23684271, SRR23684272, SRR23684273, and SRR23684274.
