# Supplementary material for: Characterization of tea (Camellia sinensis L.) flower extract and insights into its antifungal susceptibilities of Aspergillus flavus
Source: BMC Complement Med Ther. 2023 Aug 14;23:286. doi: 10.1186/s12906-023-04122-5 (PMC10424394; doi:10.1186/s12906-023-04122-5)
Supplement: Supplementary file 1 — Supplementary Material 1 [file 12906_2023_4122_MOESM1_ESM.docx]

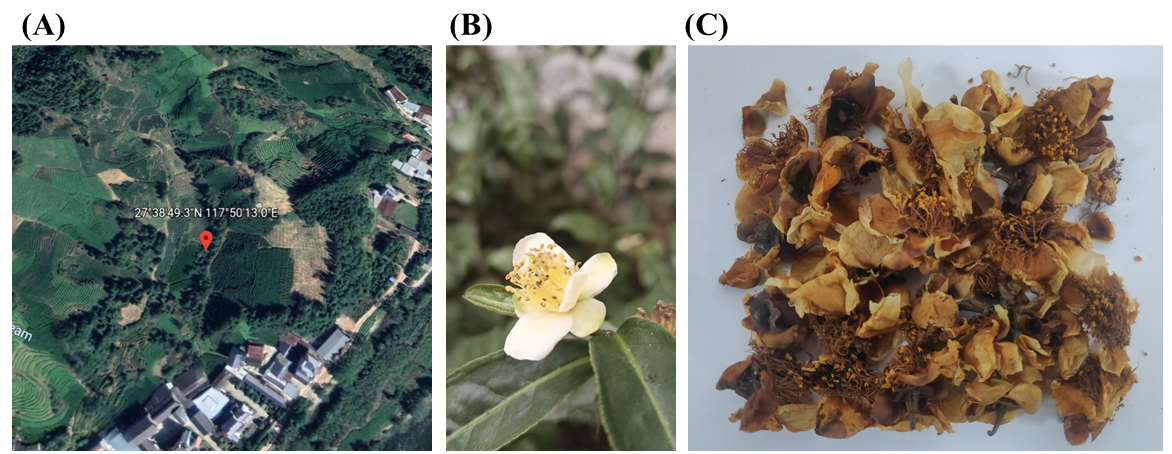


**Figure S1.** The source of tea flower. (A) Geographical map of the tea plantation (27°38′N, 117°50′E). The map was obtained from Google earth. (B) The fresh tea flower. (C) The dry tea flower by natural-air drying.
