# Supplementary material for: Characterization of tea (Camellia sinensis L.) flower extract and insights into its antifungal susceptibilities of Aspergillus flavus
Source: BMC Complement Med Ther. 2023 Aug 14;23:286. doi: 10.1186/s12906-023-04122-5 (PMC10424394; doi:10.1186/s12906-023-04122-5)
Supplement: Supplementary file 2 — Supplementary Material 2 [file 12906_2023_4122_MOESM2_ESM.docx]

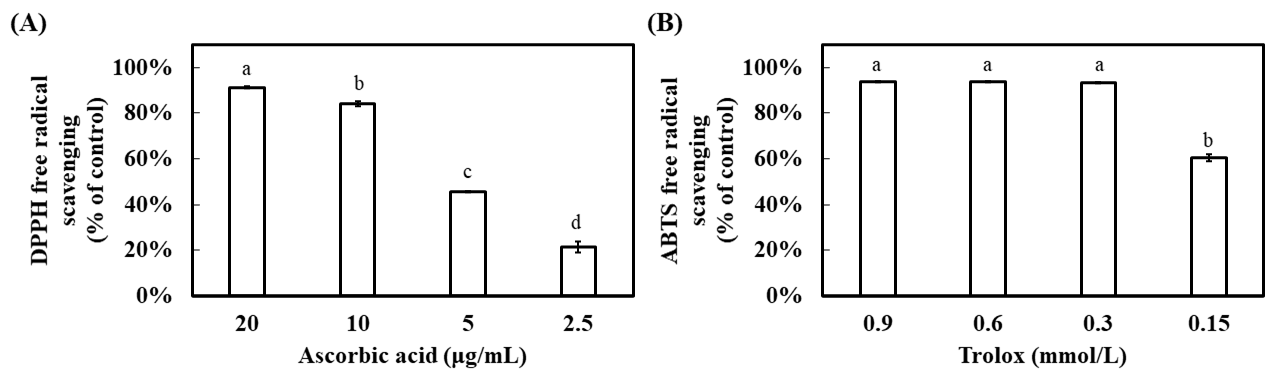


**Figure S2.** The antioxidant activity of ascorbic acid and Trolox. Free radical scavenging ability was determined by the (A) DPPH and (B) ABTS methods. Sterile distilled water was used as a control. Results are presented as mean ± S.D. (*n* = 3).
