# Supplementary figures and images for: Characterization of tea (Camellia sinensis L.) flower extract and insights into its antifungal susceptibilities of Aspergillus flavus
Source: BMC Complement Med Ther. 2023 Aug 14;23:286. doi: 10.1186/s12906-023-04122-5 (PMC10424394; doi:10.1186/s12906-023-04122-5)

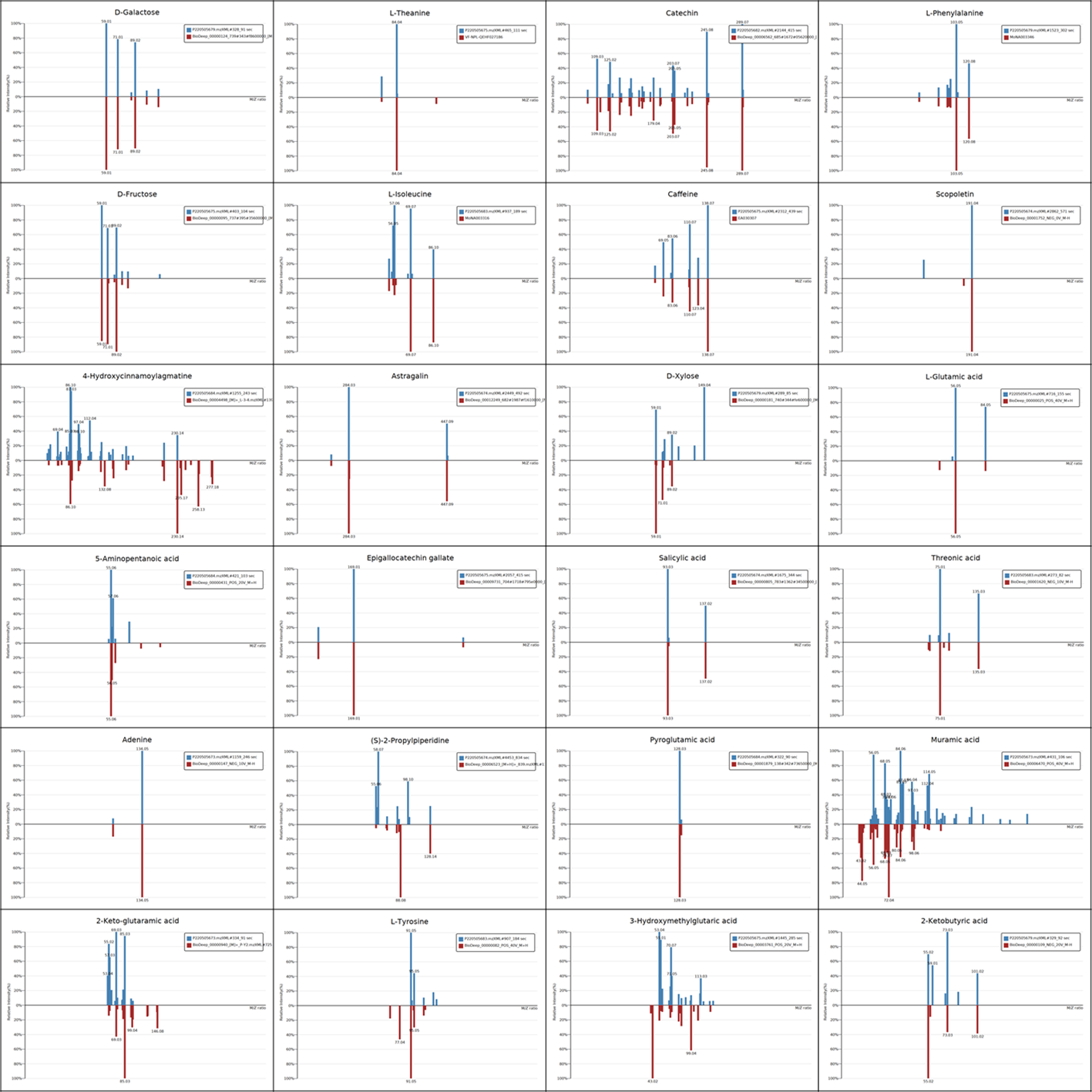


**Figure S3.** The most abundant metabolites of the TFE in MS/MS spectrum.

Supplement: Supplementary file 3 — Supplementary Material 3 [file 12906_2023_4122_MOESM3_ESM.docx]
