# Supplementary material for: Characterization of tea (Camellia sinensis L.) flower extract and insights into its antifungal susceptibilities of Aspergillus flavus
Source: BMC Complement Med Ther. 2023 Aug 14;23:286. doi: 10.1186/s12906-023-04122-5 (PMC10424394; doi:10.1186/s12906-023-04122-5)
Supplement: Supplementary file 4 — Supplementary Material 4 [file 12906_2023_4122_MOESM4_ESM.docx]

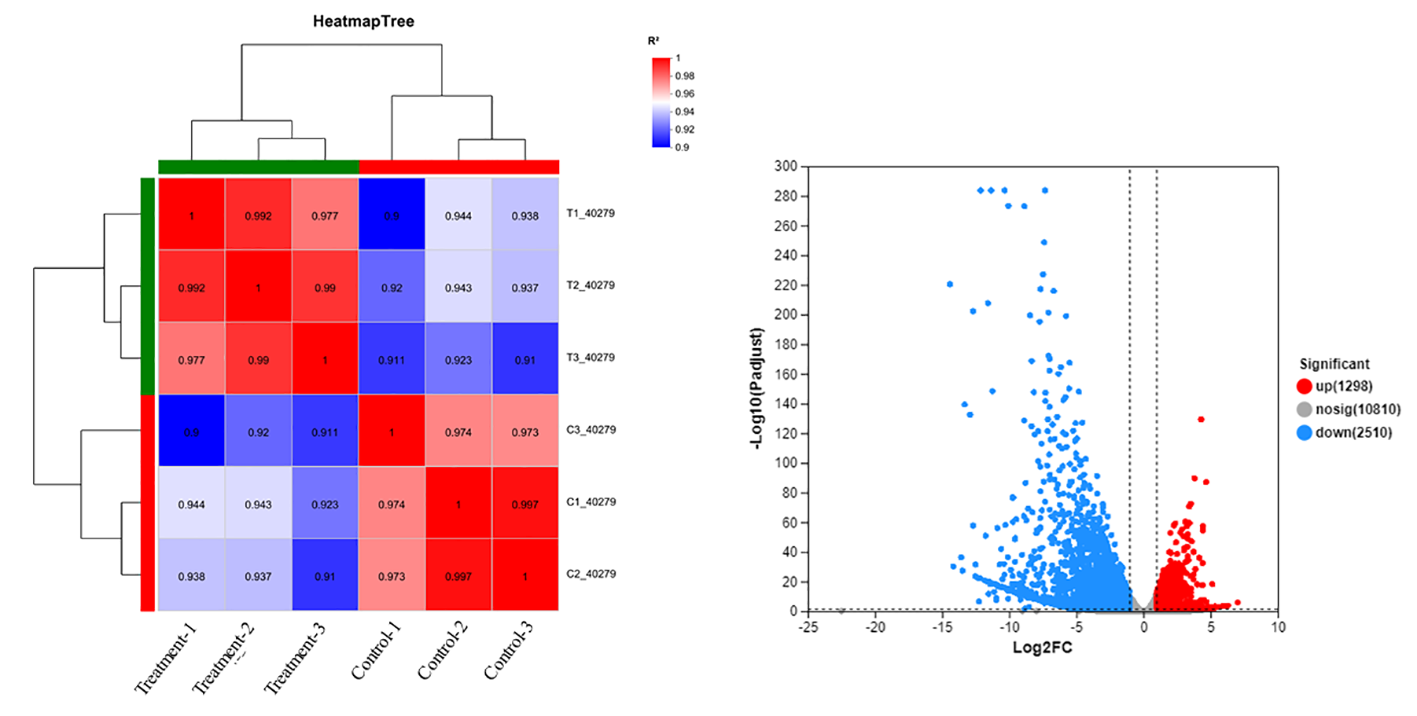


**Figure S4.** The heatmap tree and volcano plots of the DEGs by *A. flavus* CCTCC AF 2023038 treated with and without 2-ketobutyric acid. Treatment-1, Treatment-2, and Treatment-3 indicate the DEGs cluster of *A. flavus* CCTCC AF 2023038 in triplicate with the 1 mg/mL 2-ketobutyric acid treatment. Control-1, Control-2, and Control-3 indicate the DEGs cluster of *A. flavus* CCTCC AF 2023038 in triplicate without 2-ketobutyric acid treatment.
