# Supplementary material for: Characterization of tea (Camellia sinensis L.) flower extract and insights into its antifungal susceptibilities of Aspergillus flavus
Source: BMC Complement Med Ther. 2023 Aug 14;23:286. doi: 10.1186/s12906-023-04122-5 (PMC10424394; doi:10.1186/s12906-023-04122-5)
Supplement: Supplementary file 5 — Supplementary Material 5 [file 12906_2023_4122_MOESM5_ESM.docx]

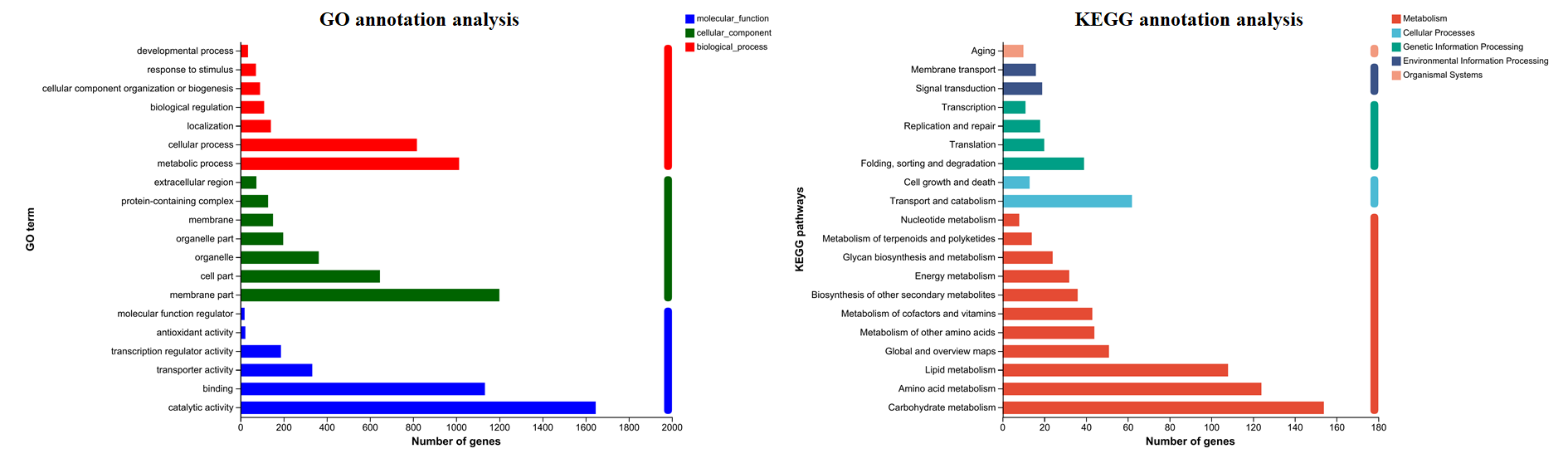


**Figure S5**. The classification of the DEGs by *A. flavus* CCTCC AF 2023038 treated with and without 2-ketobutyric acid according to the GO and KEGG databases.
