# Supplementary material for: Characterization of tea (Camellia sinensis L.) flower extract and insights into its antifungal susceptibilities of Aspergillus flavus
Source: BMC Complement Med Ther. 2023 Aug 14;23:286. doi: 10.1186/s12906-023-04122-5 (PMC10424394; doi:10.1186/s12906-023-04122-5)
Supplement: Supplementary file 6 — Supplementary Material 6 [file 12906_2023_4122_MOESM6_ESM.docx]

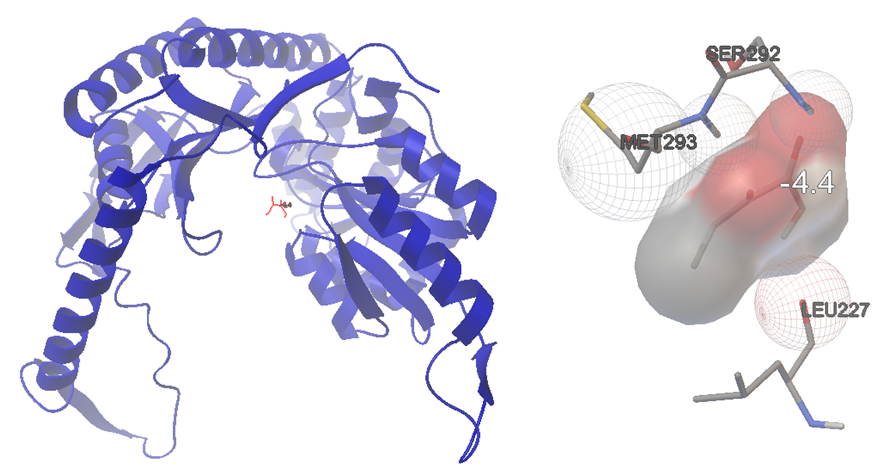


**Figure S6.** The molecular docking of 2-ketobutyric acid with serine palmitoyl-transferase (PYH86761). The protein-ligand complexes were implemented in AutoDock Vina (https://autodock.scripps.edu).
