# Supplementary material for: Characterization of tea (Camellia sinensis L.) flower extract and insights into its antifungal susceptibilities of Aspergillus flavus
Source: BMC Complement Med Ther. 2023 Aug 14;23:286. doi: 10.1186/s12906-023-04122-5 (PMC10424394; doi:10.1186/s12906-023-04122-5)
Supplement: Supplementary file 7 — Supplementary Material 7 [file 12906_2023_4122_MOESM7_ESM.docx]

**Table S1.** The primer sets were utilized for RT-qPCR.

| Gene ID | Primers |
| --- | --- |
| TRINITY_DN7498_c0_g2 | Forward: 5’ CACCTTTGTCGTTCCCCAAT 3’ |
|  | Reverse: 5’ TCTTAGCGCAGTCCACAAGC 3’ |
| TRINITY_DN7871_c0_g1 | Forward: 5’ CTGCGTTGTACCCTCACTCC 3’ |
|  | Reverse: 5’ AGTGGGACATTCCCCATTGA 3’ |
| TRINITY_DN8812_c0_g1 | Forward: 5’ TGGTTCAAGTGCATACGAAGC 3’ |
|  | Reverse: 5’ CTGTCGTTCCGACTTGTTGAG 3’ |
| TRINITY_DN10877_c0_g1 | Forward: 5’ GGCTTGAGAAGACGGATGAG 3’ |
|  | Reverse: 5’ GCTCAAGGTCGCAGAACAGA 3’ |
| TRINITY_DN119_c0_g1 | Forward: 5’ CAACCTCGAAGCTGTACGGA 3’ |
|  | Reverse: 5’ AGCTTTGCCCTCTGGCATAG 3’ |
| TRINITY_DN3275_c0_g1 | Forward: 5’ CCTTATCCGTCCATCCCTTC 3’ |
|  | Reverse: 5’ GGACATTTGAGGGGAAGTGG 3’ |
| β-Tubulin | Forward: 5’ CCATACCAGGGACGAAGAGT 3’ |
|  | Reverse: 5’ ATCACTCATTCCAACGGTGG 3’ |
