# Supplementary material for: Characterization of tea (Camellia sinensis L.) flower extract and insights into its antifungal susceptibilities of Aspergillus flavus
Source: BMC Complement Med Ther. 2023 Aug 14;23:286. doi: 10.1186/s12906-023-04122-5 (PMC10424394; doi:10.1186/s12906-023-04122-5)
Supplement: Supplementary file 8 — Supplementary Material 8 [file 12906_2023_4122_MOESM8_ESM.docx]

**Table S2.** The summary of RNA-seq data performed by an Illumina NovaSeq 6000.

| Sample | Raw bases | Clean reads | Error rate (%) | Q30 (%)^a^ | GC content (%) |
| --- | --- | --- | --- | --- | --- |
| Control-1 | 1.24E+10 | 81,435,824 | 0.0246 | 94.63 | 52.52 |
| Control-2 | 8.76E+09 | 57,553,528 | 0.025 | 94.15 | 52.33 |
| Control-3 | 8.84E+09 | 57,910,784 | 0.0247 | 94.48 | 52.65 |
| 2-Ketobutyric acid-1 | 7.96E+09 | 52,219,932 | 0.0251 | 94.07 | 52.76 |
| 2-Ketobutyric acid-2 | 9.49E+09 | 62,178,070 | 0.0248 | 94.38 | 52.71 |
| 2-Ketobutyric acid-3 | 8.71E+09 | 57,208,464 | 0.0248 | 94.41 | 52.9 |

^a^ Q30 indexed the percentage of bases whose quality is above 99.9% to the total bases.
