# Supplementary material for: Characterization of tea (Camellia sinensis L.) flower extract and insights into its antifungal susceptibilities of Aspergillus flavus
Source: BMC Complement Med Ther. 2023 Aug 14;23:286. doi: 10.1186/s12906-023-04122-5 (PMC10424394; doi:10.1186/s12906-023-04122-5)
Supplement: Supplementary file 9 — Supplementary Material 9 [file 12906_2023_4122_MOESM9_ESM.docx]

**Table S3.** The 20 most up-regulated genes responding to the ratio of the 2-ketobutyric acid treatment to control in *Aspergillus* sp. XM279.

| No. | Gene Id | NR description | FC | Log_2_FC | P-value |
| --- | --- | --- | --- | --- | --- |
| 1 | TRINITY_DN7498_c0_g2 | hypothetical protein CA14_010599 | 130.158 | 7.024121 | 2.75E-07 |
| 2 | TRINITY_DN7338_c0_g2 | benzoate 4-monooxygenase cytochrome P450 | 78.058 | 6.286481 | 4.89E-05 |
| 3 | TRINITY_DN10736_c0_g1 | － | 65.72 | 6.038266 | 0.000143 |
| 4 | TRINITY_DN9138_c0_g1 | － | 60.166 | 5.910879 | 0.000141 |
| 5 | TRINITY_DN10136_c0_g1 | － | 48.424 | 5.597659 | 0.000471 |
| 6 | TRINITY_DN11628_c0_g1 | － | 40.689 | 5.34657 | 0.001336 |
| 7 | TRINITY_DN7871_c0_g1 | － | 40.245 | 5.330726 | 0.001487 |
| 8 | TRINITY_DN11728_c0_g1 | cytochrome oxidase subunit 3 | 39.908 | 5.318597 | 0.000211 |
| 9 | TRINITY_DN4885_c0_g3 | choline dehydrogenase | 39.469 | 5.302665 | 0.00257 |
| 10 | TRINITY_DN11438_c0_g1 |  | 38.024 | 5.248841 | 0.002368 |
| 11 | TRINITY_DN8812_c0_g1 | 6-hydroxy-D-nicotine oxidase | 35.075 | 5.132374 | 4.71E-20 |
| 12 | TRINITY_DN9478_c0_g1 | uncharacterized protein | 33.082 | 5.047991 | 0.000791 |
| 13 | TRINITY_DN1232_c1_g1 | － | 32.266 | 5.011933 | 0.004486 |
| 14 | TRINITY_DN8619_c0_g1 | － | 30.814 | 4.945517 | 0.001554 |
| 15 | TRINITY_DN10259_c0_g1 | － | 30.319 | 4.922147 | 0.012515 |
| 16 | TRINITY_DN4885_c0_g1 | glucose dehydrogenase/choline dehydrogenase/mandelonitrile lyase | 28.919 | 4.853947 | 0.001214 |
| 17 | TRINITY_DN4693_c0_g2 | hypothetical protein | 28.381 | 4.82687 | 0.002864 |
| 18 | TRINITY_DN10469_c0_g1 | hypothetical protein | 27.978 | 4.806244 | 0.000443 |
| 19 | TRINITY_DN6295_c0_g3 | hypothetical protein | 27.176 | 4.76424 | 0.011925 |
| 20 | TRINITY_DN1306_c2_g1 | － | 27.027 | 4.756308 | 0.002287 |
