# Supplementary material for: Characterization of tea (Camellia sinensis L.) flower extract and insights into its antifungal susceptibilities of Aspergillus flavus
Source: BMC Complement Med Ther. 2023 Aug 14;23:286. doi: 10.1186/s12906-023-04122-5 (PMC10424394; doi:10.1186/s12906-023-04122-5)
Supplement: Supplementary file 10 — Supplementary Material 10 [file 12906_2023_4122_MOESM10_ESM.docx]

**Table S4.** The 20 most down-regulated genes responding to the ratio of the 2-ketobutyric acid treatment to control in *Aspergillus* sp. XM279.

| No. | Gene Id | NR description | FC | Log_2_FC | P-value |
| --- | --- | --- | --- | --- | --- |
| 1 | TRINITY_DN10438_c0_g1 | uncharacterized protein | 0 | -11.0989 | 7.95E-15 |
| 2 | TRINITY_DN10650_c0_g1 | hypothetical protein | 0 | -13.4766 | 1.76E-29 |
| 3 | TRINITY_DN10877_c0_g1 | uncharacterized protein | 0 | -11.624 | 5.92E-22 |
| 4 | TRINITY_DN11453_c0_g1 | unnamed protein product | 0 | -12.2614 | 3.43E-24 |
| 5 | TRINITY_DN11453_c1_g1 | hypothetical protein | 0 | -11.8147 | 8.46E-23 |
| 6 | TRINITY_DN1174_c0_g1 | glutathione transferase 1 | 0 | -11.5844 | 1.85E-211 |
| 7 | TRINITY_DN119_c0_g1 | hypothetical protein | 0 | -12.6899 | 5.38E-206 |
| 8 | TRINITY_DN1871_c0_g1 | uncharacterized protein | 0 | -11.3437 | 0 |
| 9 | TRINITY_DN2004_c0_g1 | unnamed protein product | 0 | -12.2515 | 6.48E-08 |
| 10 | TRINITY_DN2004_c0_g2 | uncharacterized protein | 0 | -10.9688 | 2.58E-10 |
| 11 | TRINITY_DN3275_c0_g1 | Imizoquin biosynthesis cluster protein F | 0 | -14.4123 | 3.05E-224 |
| 12 | TRINITY_DN3383_c0_g1 | putative cytochrome P450 | 0 | -11.043 | 3.76E-20 |
| 13 | TRINITY_DN4179_c0_g1 | CobW/HypB/UreG, nucleotide-binding domain-containing protein | 0 | -12.6883 | 2.33E-60 |
| 14 | TRINITY_DN4404_c0_g1 | uncharacterized protein | 0 | -12.5195 | 1.51E-25 |
| 15 | TRINITY_DN4498_c0_g1 | putative citrate synthase | 0 | -11.4177 | 3.78E-20 |
| 16 | TRINITY_DN5514_c0_g1 | aminotransferase | 0 | -11.4924 | 1.60E-21 |
| 17 | TRINITY_DN5692_c0_g1 | unnamed protein product | 0 | -12.9083 | 9.16E-136 |
| 18 | TRINITY_DN5839_c0_g2 | uncharacterized protein | 0 | -13.3248 | 1.45E-142 |
| 19 | TRINITY_DN6208_c0_g1 | uncharacterized protein | 0 | -12.1492 | 3.26E-24 |
| 20 | TRINITY_DN6387_c0_g1 | radH flavin-dependent halogenase | 0 | -11.7533 | 2.60E-53 |
