# Supplementary material for: Characterization of tea (Camellia sinensis L.) flower extract and insights into its antifungal susceptibilities of Aspergillus flavus
Source: BMC Complement Med Ther. 2023 Aug 14;23:286. doi: 10.1186/s12906-023-04122-5 (PMC10424394; doi:10.1186/s12906-023-04122-5)
Supplement: Supplementary file 11 — Supplementary Material 11 [file 12906_2023_4122_MOESM11_ESM.docx]

**Table S5.** Gene expression comparison including up-regulation and down-regulation by RNA-seq and real-time PCR.

| Gene ID | Annotation | Log_2_ ratio by RNA-seq | Fold change by real-time PCR |
| --- | --- | --- | --- |
| TRINITY_DN7498_c0_g2 | hypothetical protein CA14_010599 | 7.024121 | 25.0854 |
| TRINITY_DN7871_c0_g1 | － | 5.330726 | 3.07324 |
| TRINITY_DN8812_c0_g1 | 6-hydroxy-D-nicotine oxidase | 5.132374 | 4.09845 |
| TRINITY_DN10877_c0_g1 | uncharacterized protein | -11.624 | -5.93866 |
| TRINITY_DN119_c0_g1 | hypothetical protein | -12.6899 | -2.65152 |
| TRINITY_DN3275_c0_g1 | Imizoquin biosynthesis cluster protein F | -14.4123 | -6.10684 |
